# Improving the Potential for Predicting Prostate Cancer Progression in Patients on Active Surveillance Using Explainable Artificial Intelligence

**DOI:** 10.3390/cancers17223598

**Published:** 2025-11-07

**Authors:** Olga Vershinina, Nikita Sushentsev, Alexey Zaikin, Oleg Blyuss, Tristan Barrett, Mikhail Ivanchenko

**Affiliations:** 1 Research Center in Artificial Intelligence, Institute of Information Technologies, Mathematics and Mechanics, Lobachevsky State University, Nizhny Novgorod 603022, Russia; 2 Institute of Biogerontology, Lobachevsky State University, Nizhny Novgorod 603022, Russia; 3 Department of Radiology, Addenbrooke’s Hospital and University of Cambridge, Cambridge CB2 0QQ, UK; 4 Institute for Cognitive Neuroscience, University Higher School of Economics, Moscow 101000, Russia; 5 Department of Mathematics and Women’s Cancer, University College London, London WC1E 6BT, UK; alexey.zaikin@ucl.ac.uk; 6 Centre for Cancer Screening, Prevention and Early Diagnosis, Wolfson Institute of Population Health, Queen Mary University of London, London EC1M 6BQ, UK; 7 Department of Paediatrics and Paediatric Infectious Diseases, Institute of Child’s Health, Sechenov First Moscow State Medical University (Sechenov University), Moscow 119991, Russia

**Keywords:** prostate cancer, active surveillance, progression prediction, MRI-derived radiomics, machine learning, explainable artificial intelligence

## Abstract

**Background**: Approximately half of prostate cancer (PCa) patients present with low- or intermediate-risk disease eligible for active surveillance (AS). However, a substantial proportion of individuals experience pathological progression during follow-up. In this study, we developed predictive models for histopathological PCa progression in patients on AS. **Methods**: The dataset comprised patients with biopsy-confirmed PCa and a minimum follow-up of two years. All patients underwent regular surveillance, including prostate-specific antigen (PSA) measurements and MRI examinations. Each patient had three to six consecutive MRI scans available for analysis. Histopathological progression was defined as an upgrade to a higher grade group on repeat targeted biopsy. Predictive modeling integrated radiomic and clinical variables using machine learning (ML). SHapley Additive exPlanations (SHAP) was used for feature interpretation. **Results**: Three models were obtained: (1) a baseline model utilizing radiomic features from initial MRI scans combined with baseline PSA density (PSAd) (AUC = 0.793, sensitivity = 0.690, specificity = 0.830); (2) a delta model incorporating feature changes between latest and baseline available MRI scans with final PSAd (AUC = 0.913, sensitivity = 0.793, specificity = 0.936); and (3) a time series model analyzing the complete series of radiomic features and PSAd (AUC = 0.917, sensitivity = 0.828, specificity = 0.894). **Conclusions**: Our predictive models demonstrated strong performance in distinguishing progressors from non-progressors, suggesting that radiomic analysis combined with ML has significant potential to enhance PCa management. This approach could enable more personalized treatment strategies and improve clinical decision-making for patients undergoing AS.

## Simple Summary

This study aimed to develop predictive models for prostate cancer (PCa) progression under active surveillance (AS). Accurate progression risk stratification could significantly improve clinical outcomes while reducing the requirement for repeated invasive biopsies. We demonstrated significantly enhanced prognostic performance through the integration of MRI-derived radiomic features with prostate-specific antigen density (PSAd), complemented by explainable artificial intelligence (XAI) for feature selection. Our findings suggest substantial potential for improving detection of progression to clinically significant PCa and clinical management.

## 1. Introduction

Prostate cancer (PCa) is the second most frequently diagnosed malignancy and the fifth leading cause of cancer-related mortality among men worldwide [1]. However, due to widespread prostate-specific antigen (PSA) screening, nearly half of all men are diagnosed with the disease at an early stage and have a favorable, low-risk prognosis [2]. Active surveillance (AS) is the recommended management strategy for these patients, entailing long-term monitoring and permitting the deferral of radical treatment in the absence of disease progression [3]. Cancer registry datasets confirm that AS is a safe management approach, with 49% of patients being progression-free at 10 years and only a 0.1% rate of prostate cancer-specific mortality [4]. Several studies have confirmed that AS in patients with localized PCa is not inferior to initial treatment in terms of survival and life expectancy [5,6]. Patients managed with AS generally maintain good health without significant psychological detriment in the short term [7], whereas surgery is associated with a higher rate of adverse events [5]. Nevertheless, histopathological progression and disease reclassification occur in an estimated 22–38% of men during AS [8,9,10,11,12,13,14,15]. Thus, reliable clinical tools are critically needed for both baseline risk stratification and the continuous reassessment of PCa progression risk.

Despite a lack of standardization in AS implementation—particularly in the timing of examinations and threshold values [16]—surveillance protocols typically share a common core of PSA testing, digital rectal examination, and repeat prostate biopsies. Prostate biopsy is an invasive procedure that causes patient discomfort and carries risks of bleeding and infection [17,18]. Consequently, researchers are investigating non-invasive methods, particularly magnetic resonance imaging (MRI), for monitoring patients on AS [19,20,21,22,23]. However, serial MRI for patients on AS has a low positive predictive value—partly due to the subjective, reader-dependent nature of the assessment—and cannot yet replace biopsy [24].

These limitations have spurred the development of radiomics, which extracts quantitative features of intensity, shape, and texture from medical images that are imperceptible to visual assessment. The analysis of multidimensional radiomics data commonly relies on machine learning (ML) and artificial intelligence (AI) algorithms. A significant gap exists in radiomics research for PCa: while many prognostic models have been developed, few address the prediction of progression in patients managed with AS. Studies [25,26] have evaluated the value of MRI-derived radiomic features for the baseline prediction of disease progression in patients undergoing AS. In studies [27,28], classifiers were developed to predict PCa progression during AS using delta-radiomic features. Finally, recurrent neural networks [29] have also been employed to predict PCa progression during AS, utilizing time series changes in tumour-derived radiomic features and PSA density (PSAd). The listed predictive models demonstrated good performance, with area under the receiver operating characteristic curve values ranging from 0.75 for baseline models to 0.86 for the model incorporating time series data.

In this study, we aimed to improve prediction of PCa progression during AS by developing three distinct models: baseline prediction, prediction based on delta features, and time series-based prediction. To achieve this, we evaluated a wide range of ML algorithms and used SHapley Additive exPlanations (SHAP), an explainable AI approach, to interpret the models and select the most important predictive features. Results indicate that our models—utilizing radiomics and PSAd—surpass existing models, while the success of the ensemble model underscores the potential of integrating data from different observations. Ultimately, this work enhances progression prediction, paving the way for personalized monitoring strategies in low-risk PCa.

## 2. Materials and Methods

### 2.1. Study Population

This retrospective exploratory study included consecutive patients with biopsy-proven PCa enrolled on AS in Cambridge University Hospitals (CUH) NHS Foundation trust between November 2012 and February 2019. To be enrolled, patients were required to have International Society of Urological Pathology (ISUP) grade group 1 or 2 disease (with ≤10% Gleason pattern 4), along with at least one MRI-visible lesion, a minimum 2-year follow-up, three 3T MRI scans on the same magnet, and one repeat targeted biopsy within 12 months of the final MRI. Patients were excluded if they had undergone any prior treatment for PCa or benign disease, or had a total hip replacement or other pelvic metalwork. Of the 364 patients with PCa in the AS program, 76 were selected for this study. These patients were divided into two groups according to disease progression status. PCa progression (n = 29) was defined by a switch to radical treatment, which was prompted by confirmed histopathological progression (an increase in ISUP grade group from the diagnostic biopsy) on a repeat targeted biopsy.

### 2.2. Targeted Biopsy Protocol

Targeted biopsies were performed by three expert urologists (more than 10 years of experience) using MRI/ultrasound fusion. The approach—either transrectal (DynaCAD, InVivo Corp, Orlando, FL, USA) or transperineal (Biopsee, Oncology Systems Limited, Shrewsbury, UK)—was determined by clinical recommendation. Twelve systematic cores were taken as part of the transrectal procedures, and 24 systematic cores were obtained during transperineal approach, following the Ginsburg protocol [30]. In addition, 2–4 targeted cores were sampled from each lesion identified on MRI, as previously described [31]. Repeat biopsies were performed either at protocol-specified intervals (12 and 36 months after AS enrollment) or were triggered by clinical concerns. These included three consecutive elevations of PSA above a predefined threshold or radiological progression (a PRECISE score ≥ 4) [19].

### 2.3. MRI Acquisition Parameters

All patients underwent prostate MRI on a 3T MR750 scanner (GE Healthcare, Waukesha, WI, USA) using a 32-channel receiver coil. The full imaging protocol is detailed in Supplementary Table S1. The baseline examination consisted of a multiparametric MRI protocol that included multiplanar high-resolution T2-weighted imaging (T2WI), diffusion-weighted imaging (DWI), and dynamic contrast-enhanced (DCE) MRI. Apparent diffusion coefficient (ADC) maps were calculated using DWI. Subsequent follow-up scans utilized a biparametric MRI protocol, which omitted DCE-MRI. Unless clinically contraindicated, patients received an intravenous injection of 20 mg/mL hyoscine butylbromide (Buscopan; Boehringer, Ingelheim am Rhein, Germany) prior to imaging to reduce bowel peristalsis.

### 2.4. Image Segmentation and Radiomic Feature Extraction

Tumor regions of interest (ROIs) were delineated on de-identified anatomical T2WI and ADC maps by two readers: a urogenital radiologist and a research fellow, both with substantial experience in prostate MRI. Segmentations were performed by consensus using ITK-SNAP [32]. Radiomic features were extracted from T2WI and ADC maps using the PyRadiomics package v2.0 [33] in Python v3.7. To assess feature robustness, the following strategies were used: applying ROI morphological perturbations to exclude features susceptible to segmentation variability [34]; evaluating the association between features and MRI acquisition parameters; and implementing an intraclass correlation coefficient (ICC) threshold of >0.8 to select highly robust features. A more detailed description of the robustness analysis of radiomic features is provided in previous works [25,27,29] and in Supplementary Materials. Further predictive modeling incorporated only features that were robust across all time points for all patients, resulting in 17 features from T2WI and 27 from ADC (Supplementary Table S2).

### 2.5. Data Preprocessing

Three data types were analyzed:

• Baseline features derived from the initial observation;
• Delta features, calculated as the arithmetic difference between the final and baseline observations;
• Time series of features, incorporating all available examinations.

Alongside texture features (T2WI- and ADC-derived radiomics), non-radiomic clinical parameters were added, namely PSA and PSAd. PSAd, calculated as the ratio of PSA to MRI-derived prostate volume, is a well-established independent predictor used in clinical practice [35,36,37,38]. All obtained quantitative features were standardized by subtracting the mean and dividing by the standard deviation. To handle variable-length time series, a post-padding procedure was applied after the standardization step.

### 2.6. Predictive Modeling

The prediction of histopathological PCa progression was formulated as a binary classification task, with class 1 representing progressors and class 0 representing non-progressors. Due to the limited cohort size, models were evaluated using leave-one-out cross-validation (LOOCV) [39] instead of a hold-out train-test split. LOOCV is a method where the model is repeatedly trained on all data points except one, which is used for validation, and the final performance estimate is obtained by averaging the resulting quality metrics across all validation iterations. The balanced accuracy, accounting for class imbalance, served as the primary metric to assess classification quality. Furthermore, both the F1-score and the Area Under the Receiver Operating Characteristic Curve (AUC) were computed.

Predictive models using tabular data (including baseline and delta features) were constructed with traditional and advanced ML algorithms. Among them were k-Nearest Neighbors (kNN) [40], Logistic Regression (LR) [41], Support Vector Machine (SVM) [42], Decision Tree (DT) [43], Random Forest (RF) [44], Gradient Boosting (GB) [45], eXtreme Gradient Boosting (XGBoost) [46], Light Gradient Boosted Machine (LightGBM) [47], and Category Boosting (CatBoost) [48].

Time series classification was performed using a Long Short-Term Memory (LSTM) architecture [49]. To handle variable sequence lengths, a masking layer was applied to the network input. The LSTM cell utilized a sigmoid activation function for the recurrent steps and a hyperbolic tangent (tanh) function for the cell state and output. The model’s weights were initialized with specific schemes to promote stable training: the input kernel weights were initialized using the Glorot uniform scheme, and the recurrent kernel weights were initialized with a random orthogonal matrix. All bias vectors were initialized to zero. For regularization, dropout was applied to the input and recurrent connections of the LSTM layer to mitigate overfitting. The output from the LSTM was then passed through a batch normalization layer and, finally, a densely connected output layer with a single neuron and a sigmoid activation function to generate prediction probabilities. The resulting neural network was trained using the Adam optimization algorithm.

The hyperparameters of all models were optimized using the Tree-structured Parzen Estimator (TPE) algorithm [50,51], implemented in the Optuna package [52]. The TPE is a Bayesian optimization algorithm that models the distribution of promising hyperparameters to efficiently explore the search space and focus on regions most likely to improve performance. The total number of optimization trials per experiment was set to 100. The optimal set of hyperparameters was selected from the trial that achieved the maximum balanced accuracy under the LOOCV scheme. The full set of tunable hyperparameters, their descriptions, and their respective search distributions are detailed in Supplementary Table S3.

The Shapley Additive exPlanations (SHAP), a model-agnostic approach based on cooperative game theory [53], was utilized to interpret the final prognostic models. This method quantified feature contributions to predictions, thereby identifying influential features and enhancing comprehension of the underlying mechanisms involved in PCa progression prediction. For ML models using tabular data, the universal Explainer from the SHAP package was employed, while the GradientExplainer was utilized for the LSTM architecture.

Training and evaluation of predictive models based on both baseline and delta features were conducted using Python 3.11 with the following packages: scikit-learn v1.6.1, XGBoost v3.0.0, LightGBM v4.6.0, CatBoost v1.2.8, Optuna v4.2.1, and SHAP v0.47.1. Due to version incompatibilities, the LSTM model was developed in a separate environment using Python 3.7, TensorFlow v1.15.5, Optuna v4.0.0, and SHAP v0.42.1.

### 2.7. Statistical Analysis

Group comparisons for continuous variables were performed with the Mann–Whitney U test, while categorical variables were assessed with the chi-square test. A two-sided *p*-value < 0.05 was considered statistically significant.

### 2.8. Workflow of the Study

**Figure 1:**
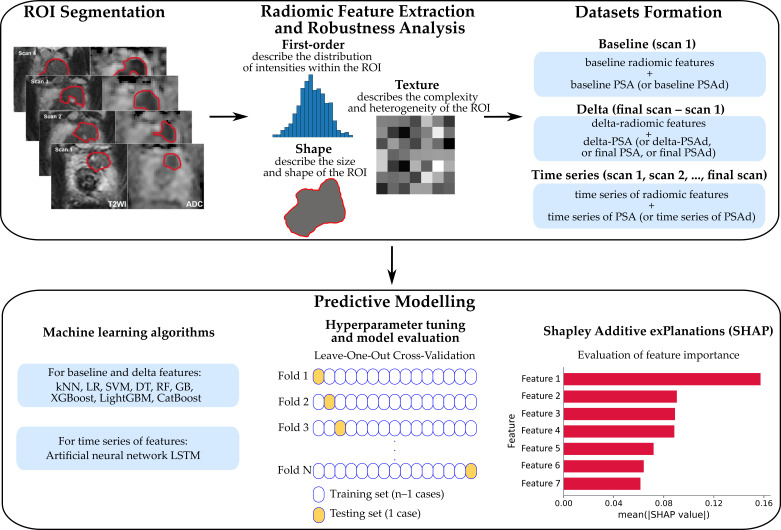
The workflow illustrating the development of models for predicting PCa progression.

The overall workflow for developing models to predict histopathological PCa progression for patients on AS is shown in Figure 1. The methodology algorithm consists of the following steps:

1. ROI segmentation on T2WI and ADC maps (Section 2.4);
2. Extraction of radiomic features (first-order, shape, and texture) and further analysis of their robustness (Section 2.4);
3. Formation of datasets depending on the scans and features considered (Section 2.5);
4. Predictive modeling for various datasets, including the consideration of different ML algorithms, tuning of hyperparameters via LOOCV, selection of the best models, and their interpretation and improvement using SHAP (Section 2.6).

## 3. Results

### 3.1. Patient Characteristics

This study included a cohort of 76 PCa patients managed under AS. The resulting number of consecutive MRI scans per patient varied: 31 patients had three scans, 25 had four, 16 had five, and 4 had six. During follow-up, 29 patients (38.2%) demonstrated histopathological disease progression (class 1), and 47 patients (61.8%) had stable disease (class 0). Baseline clinicopathological characteristics of the patients included age, gland volume derived from MRI, PSA and PSAd values, ISUP (International Society of Urological Pathology) grade group, and the PI-RADS (Prostate Imaging Reporting and Data System) score. The patients studied had ISUP groups 1 and 2, determined by biopsy results. Grade group 1 (Gleason score 3 + 3 = 6) is characterized by discrete, well-formed glands and represents the most indolent form of prostate cancer, associated with a favorable prognosis. Grade group 2 (Gleason score 3 + 4 = 7) represents an intermediate stage where a higher-grade component is introduced, signifying a shift toward more aggressive biological potential. Patients selected for this study had PI-RADS scores of 3, 4, or 5 based on MRI. According to this scoring system, a PI-RADS 3 lesion is considered of intermediate risk for clinically significant prostate cancer (csPCa), whereas a PI-RADS 5 lesion signifies a very high risk. We performed statistical analyses to compare baseline characteristics and follow-up time between progressors and non-progressors; the results are presented in Table 1. Compared to non-progressors, progressors had a significantly lower baseline gland volume (*p* = 0.005) and a significantly higher PSAd (*p* = 0.007). A statistically significant difference in PI-RADS scores was also observed (*p* = 0.021). The progression group contained a higher proportion of PI-RADS 5 lesions and a lower proportion of PI-RADS 3 lesions. No statistically significant differences were observed between progressors and non-progressors in baseline age, PSA, and biopsy ISUP grade group. The follow-up time on AS, which had a median of 42 months, also showed no statistically significant difference.

### 3.2. Progression Prediction Models Based on Baseline Features, Delta Features, and Time Series of Features

Following the workflow presented in the Materials and Methods, after segmentation of the ROIs in the MRI, radiomic features were extracted. These included first-order features, describing the distribution of voxel intensities; shape features, quantifying the ROI’s size and geometry; and texture features, characterizing its internal complexity and heterogeneity. A robustness analysis was then performed, resulting in 17 T2WI- and 27 ADC-derived radiomic features being retained for predictive modeling (Supplementary Table S2).

Next, we constructed multiple datasets, systematically varying the data according to two parameters:

1. Temporal Context. Features were calculated from:
  (I) The baseline (initial) observation (Baseline features);
  (II) The difference between the final (last) and baseline observations (Delta features);
  (III) All available observations (Time series of features).
2. Feature Subset. For each temporal context, we created three feature subsets:
  (A) Radiomic features from T2WI and ADC maps;
  (B) Radiomic features and PSA value;
  (C) Radiomic features and PSAd value.

To the delta-radiomic features, we also added either the final PSA value (II.D) or the final PSAd value (II.E).

For each of the eleven datasets, we tuned, trained, and evaluated corresponding ML models. This included nine algorithms for baseline and delta datasets and the LSTM network for the time series data. All models underwent hyperparameter optimization via LOOCV, with balanced accuracy as the primary performance metric. The results for the optimal models are presented in Table 2. For the baseline and delta feature datasets (I and II), Table 2 displays the best-performing model from the nine evaluated. Comprehensive results for all models are provided in Supplementary Tables S4 and S5, respectively.

As can be seen, the optimal model for predicting progression using baseline data leveraged a combination of radiomic features and PSAd. (Table 2, dataset I.C). In the context of delta-radiomics, the model performance was better when the final PSAd value was included as an additional feature (Table 2, dataset II.E). Interestingly, gradient boosting (GB) was found to be the most effective ML algorithm for both baseline and delta datasets, but the optimal hyperparameter values for the two models differed. As for the time series-based models, the combination of radiomic features and PSAd yielded optimal performance (Table 2, dataset III.C). For clarity, the selected optimal model-dataset combinations will be referred to as the baseline model, delta model, and time series model in the following discussion.

### 3.3. Explainable Artificial Intelligence

To interpret the selected models, we evaluated feature importance using the SHAP approach. This method decomposes a model’s prediction for a single instance into additive feature contributions, showing how each feature pushes the base (average) prediction toward a specific outcome. A larger absolute SHAP value indicates a greater influence of the feature on the prognosis. To assess overall feature importance, we averaged the absolute SHAP values across all instances (and all time points for time series). The results showed that many features had negligible impact on the model’s predictions, demonstrating little to no importance (Supplementary Figures S1–S3). Interestingly, the progression from baseline to time series model showed a decrease in the number of features with zero importance scores. In particular, the number of such features was 36 in the baseline model (Supplementary Figure S1) and 9 in the delta model (Supplementary Figure S2). In the time series model, all features had non-zero importance, but the importance distribution also showed a gradual decay to near-zero for the least contributory ones (Supplementary Figure S3). Features with near-zero importance scores, indicating no significant relationship with the target variable, are prime candidates for removal to simplify the models and enhance predictive performance. To this end, we retrained and evaluated the models on subsets comprising the top 1, 2, …, m most important features, where m is the number of features with non-zero importance. The results of the conducted feature selection are presented in Table 3.

Dimensionality reduction via the SHAP approach significantly increased the prediction accuracy of all three models. The lowest predictive quality was observed for the baseline model (Table 3, I.C). Conversely, the model incorporating delta-radiomics and the final PSAd value (Table 3, II.E) and the time series model (Table 3, III.C) demonstrated equivalent performance, both outperforming the baseline.

Sensitivity and specificity analyses were performed on the resulting reduced models (Table 4). The time series classification model demonstrated the highest sensitivity (82.8%), indicating its high ability to identify progressors. The delta-radiomics model, on the other hand, demonstrated the highest specificity (93.6%), indicating its high ability to identify non-progressors. The baseline model had low sensitivity (69%) but good specificity (83%).

**Figure 2:**
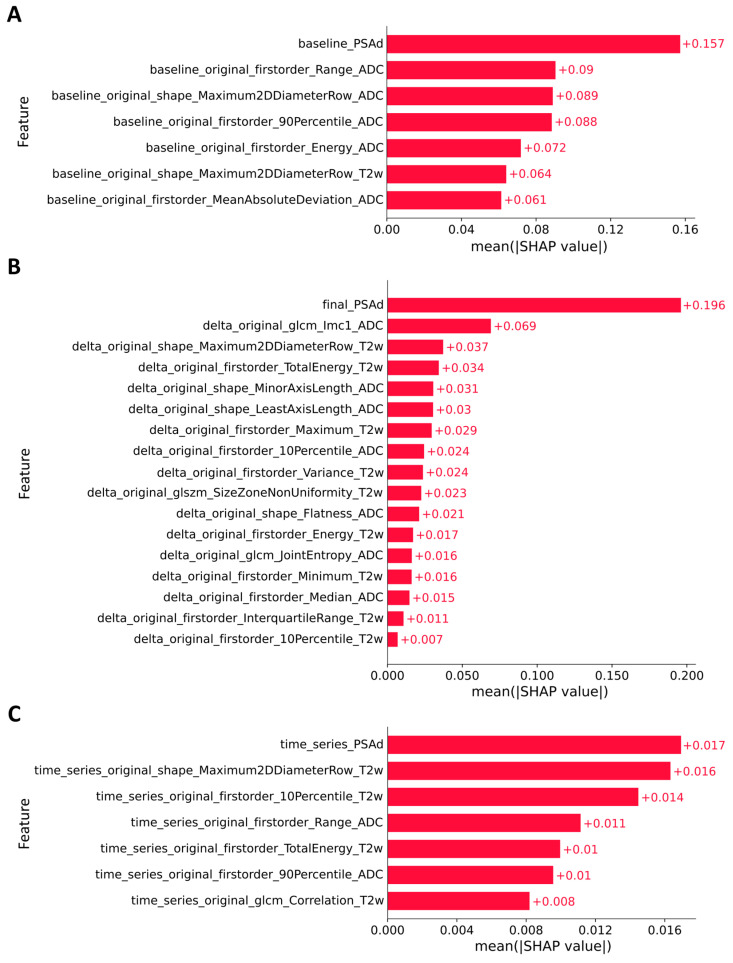
Average impact (the mean absolute SHAP values) of features on models predictions. Features are sorted by descending importance (top: most important). (**A**) Predictive model based on baseline radiomic features and baseline PSAd. (**B**) Predictive model based on delta-radiomic features and final PSAd. (**C**) Predictive model based on time series of radiomic features and time series of PSAd.

We then analyzed the SHAP feature importance of the reduced models. In Figure 2, features for each model are presented in descending order of this computed importance, from top to bottom. In the baseline model (Figure 2A), the most significant predictor is PSAd, followed by first-order (“firstorder_Range_ADC”) and shape (“shape_Maximum2DDiameterRow_ADC”) radiomic features. The first-order features quantify the distribution of voxel intensities within the tumor. In particular, the feature “firstorder_Range_ADC”—calculated as the difference between the maximum and minimum ADC values—quantifies the spread of values within the ROI, serving as an important metric of tissue heterogeneity. The shape feature—the maximum 2D tumor diameter—quantifies the largest pairwise Euclidean distance between tumor surface mesh vertices. It is noteworthy that, with one exception, all radiomic features in the model were obtained from ADC maps. These maps quantify water molecule density and mobility within tissue, and lower ADC values are a recognized indicator of more aggressive tumors. Consequently, these results underscore the critical importance of ADC maps for baseline patient diagnosis.

The delta model incorporates a larger number of features than the baseline model (Figure 2B). Notably, the second most important predictor after PSAd is the radiomic feature “glcm_Imc1_ADC”—a complex texture indicator that reflects the degree of linear relationship between adjacent pixels and takes into account the texture’s information richness. This highlights that textural changes between the final and baseline MRI scans, such as the development of fibrotic or necrotic areas, are key predictors of PCa progression during AS. The model also contains numerous first-order and shape features derived from T2WI and ADC maps. While first-order delta features describe changes in tumor signal intensity, shape delta features capture changes in its geometric properties.

In the time-series model (Figure 2C), the most important characteristics are PSAd and the maximum 2D tumor diameter (“shape_Maximum2DDiameterRow_T2w”), followed by first-order radiomic features and the texture feature “glcm_Correlation_T2w”. The last feature measures the linear correlation of brightness between adjacent pixels, serving as an indicator of tissue homogeneity; a decrease in this correlation over time may signal tumor growth and progression.

Feature analysis revealed considerable overlap across the three models. Specifically, PSAd and the maximum 2D tumor diameter (“shape_Maximum2DDiameterRow_T2w”) were identified as robust predictors in all three models, underscoring their importance from baseline to time series analysis. Furthermore, the baseline and time series models shared two common first-order statistics from the ADC map: the range of gray values in the ROI (“firstorder_Range_ADC”) and the 90th percentile (“firstorder_90Percentile_ADC”). In addition, two T2WI-derived features—the total energy (“firstorder_TotalEnergy_T2w”) and the 10th percentile (“firstorder_10Percentile_T2w”)—were common to both the delta-radiomics and time series models.

### 3.4. Combination of Predictions

The delta-radiomics model’s high specificity and positive predictive value (PPV) complement the high sensitivity and negative predictive value (NPV) of the time series model, suggesting a possible synergy between different approaches to progression assessment. We performed an additional experiment in which we averaged the progression probabilities generated by the three final models. Patient-level predictions were obtained within a LOOCV scheme. This ensemble method demonstrated significantly improved performance: balanced accuracy = 0.91, F1-score = 0.893, AUC = 0.947, sensitivity = 0.862, specificity = 0.957, PPV = 0.926, and NPV = 0.918. These findings underscore the importance of utilizing all available data types and the need for the further development of combined predictive models.

## 4. Discussion

In this study, we developed ML models utilizing MRI-derived radiomic features and PSAd to predict PCa progression in patients under AS. Three distinct data paradigms were evaluated: baseline measurements, delta features representing changes between timepoints, and complete time series of features. For each paradigm, feature selection via SHAP analysis substantially enhanced predictive performance.

Our baseline model for predicting PCa progression during the initial stage of AS utilized a GB algorithm, incorporating six radiomic features from baseline MRI scans combined with baseline PSAd. The model demonstrated balanced accuracy of 0.76, F1-score of 0.702, and AUC of 0.793. With sensitivity and specificity of 0.690 and 0.830 respectively, the model showed strong performance in identifying low-risk patients who could potentially follow less intensive surveillance protocols.

The second model employed the same GB algorithm but incorporated delta radiomics—defined as the difference in values for 16 radiomic features between final and baseline MRI scan—along with the final PSAd. This model significantly outperformed the baseline approach, achieving a balanced accuracy of 0.865, F1-score of 0.836, AUC of 0.913, sensitivity of 0.793, and specificity of 0.936. Similar to the baseline model, the delta radiomics approach maintained high specificity.

The third model utilized a LSTM recurrent neural network to analyze time series of six radiomic features and PSAd values for predicting histopathological progression during AS. The model achieved a balanced accuracy of 0.861, F1-score of 0.828, AUC of 0.917, sensitivity of 0.828, and specificity of 0.894. While the time series approach demonstrated comparable overall performance to the delta radiomics mode—expected given that the final timepoint coincided with progression assessment—it exhibited superior sensitivity in identifying high-risk patients requiring intervention.

Compared to existing radiomics-based models for predicting PCa progression during AS [25,26,27,28,29], as well as models relying solely on clinicopathological data [54,55], our models demonstrated superior performance. In direct comparisons using the same dataset, our baseline, delta radiomics, and time series models achieved AUC values of 0.793, 0.913, and 0.917, respectively, compared to previously reported values of 0.75 [25], 0.816 [27], and 0.86 [29]. These gains in predictive accuracy can be primarily attributed to our implementation of explainable AI (SHAP) for feature analysis and selection. To our knowledge, SHAP has not been previously applied to studies of PCa progression in AS. However, it is widely established for interpreting ML models, having been used for the early diagnosis of PCa [56], prediction of clinically significant PCa [57,58], pathology grade prediction [59,60,61,62], and assessment of extraprostatic extension in PCa [63].

Feature importance analysis in our models showed that PSAd is the most important characteristic for predicting progression in all models. PSAd is a well-established metric for monitoring patients with PCa and has been identified as a significant predictor of subsequent disease progression at both initial and repeat biopsies in patients on AS [8,11,12,64]. Key features in our predictive models also included first-order radiomics, which quantify the distribution of voxel intensities within the ROI, and shape-based radiomics, which describe the geometric properties of the tumor. The maximum 2D tumor diameter (“shape_Maximum2DDiameterRow_T2w”) was also included in all three models. This characteristic estimates the longest straight-line distance between two points on the 2D tumor border. Prior research has demonstrated a statistically significant increase in maximum tumor diameter on T2WI among progressors compared to non-progressors [65]. Besides, the Maximum2DDiameterRow derived from positron emission tomography (PET) imaging was one of the most significant features for predicting postsurgical Gleason scores in patients with primary PCa [66]. Additionally, the Maximum2DDiameterRow from ADC was important for predicting peripheral lymph node involvement in PCa [67]. Unlike the baseline model, which included simple first-order radiomic and shape features, the delta and time-series models incorporate additionally more complex gray-level co-occurrence matrix (GLCM) features, which describe the spatial relationships between pixels (i.e., tumor texture). These features enable the assessment of changes in the tumor’s microscopic heterogeneity and structural complexity. The prognostic value of such GLCM-based features not only for distinguishing cancerous from non-cancerous tissue but also for differentiating PCa aggressiveness is also supported by previous studies [68]. Regarding the imaging sequences used, the delta and time series models incorporated features from both T2WI and ADC maps. In contrast, the baseline model relied almost exclusively on ADC-derived features. Thus, our results underscore the crucial role of ADC maps in predicting PCa progression at the start of AS.

To evaluate the potential of multi-context data fusion for progression prediction, we constructed an ensemble model by simply averaging the probabilities from the three individual models. This ensemble proved superior, attaining a balanced accuracy of 0.91, F1-score of 0.893, AUC of 0.947, sensitivity of 0.862, and specificity of 0.957. These results highlight the critical value of incorporating data from different time contexts. A promising direction for further research would be the development of a universal model that inherently integrates all data types, which may yield even greater predictive power than the ensemble approach.

Despite the promising diagnostic potential of the developed models, our study has several limitations. The cohort was relatively small (n = 76) due to strict inclusion criteria mandating MRI-visible lesions, a condition present in only about 50% of active surveillance patients [38]. At the same time, the use of pre-biopsy prostate MRI means that contemporary AS programmes include a higher proportion of patients with MRI-visible lesions [69]. Such patients are known to have an overall higher risk of progression, which makes the case for more accurate predictive models in this context. Furthermore, the single-center design may limit the generalizability of our findings. Access to a larger, multicenter cohort would enable more robust model validation. Given the limited sample size, model performance was evaluated using LOOCV rather than a holdout test dataset. Besides, the absence of an independent external validation set makes it impossible to conclusively demonstrate that the models are robust to overfitting. A further limitation is that our analysis was restricted to PSA (and its density) and radiomic features. Future studies should therefore incorporate a broader range of clinicopathological predictors. Finally, our study did not include an analysis of feature correlations to address multicollinearity, which is a recognized method for improving ML model robustness. We have identified this fact as a limitation and suggest it be explored in future research.

## 5. Conclusions

In summary, we successfully predicted histopathological PCa progression in patients on AS by integrating MRI-derived radiomic features with PSAd. We developed three ML models leveraging data from different time contexts: baseline, delta (change between final and baseline scans), and a full time series. The delta model achieved the highest specificity, whereas the time series model showed the highest sensitivity. Using SHAP analysis, we identified the most influential features for these predictions. These results underscore the significant potential of combining radiomics with clinical variables to advance personalized medicine, enabling treatment strategies tailored to individual patient profiles. The developed predictive models can potentially be integrated into the clinical process, both at initial diagnosis and during AS, to stratify patients into progression risk groups using MRI and PSAd data, thereby reducing reliance on multiple invasive biopsies. However, before these models can be applied in real-world settings, key limitations must be addressed—namely, the small sample size, single-center design, and lack of external validation—as these factors impact models’ reliability and generalizability. Future research will aim to overcome these challenges.

## Figures and Tables

**Table 1:** Baseline clinicopathological characteristics and follow-up time of the study cohort. Characteristics are expressed as median (interquartile range) for continuous variables and as number (percentage) for categorical variables.

Variable	Total Cohort (n = 76)	Progressors (n = 29)	Non-Progressors (n = 47)	*p* (Progressors vs. Non-Progressors)
Age, years	66 (61–69)	66 (60–69)	66 (61.5–69)	1.0
Gland volume, mL	44.75 (36.0–70.0)	41.0 (29.0–47.0)	55.0 (39.5–80.5)	**0.005**
PSA, ng/mL	5.04 (3.62–7.42)	5.63 (4.02–7.7)	4.51 (3.35–7.15)	0.255
PSAd	0.10 (0.07–0.16)	0.12 (0.08–0.27)	0.09 (0.06–0.12)	**0.007**
Biopsy ISUP grade group				0.726
1	58 (76.3%)	21 (72.4%)	37 (78.7%)	
2	18 (23.7%)	8 (27.6%)	10 (21.3%)	
PI-RADS				**0.021**
3	16 (21.1%)	2 (6.9%)	14 (29.8%)	
4	26 (34.2%)	9 (31%)	17 (36.2%)	
5	34 (44.7%)	18 (62.1%)	16 (34%)	
AS follow-up time, months	42 (32.5–63.25)	40 (33–49)	43 (30.5–67.5)	0.233

Statistically significant *p*-values are highlighted in bold.

**Table 2:** Performance of optimal models for predicting histopathological PCa progression on AS. Classification quality metrics were calculated using the LOOCV scheme.

Dataset	Optimal Model	Balanced Accuracy	F1-Score	AUC
I.A	XGBoost	0.695	0.6	0.623
I.B	CatBoost	0.659	0.571	0.662
**I.C**	**GB**	**0.719**	**0.642**	**0.704**
II.A	LightGBM	0.764	0.704	0.817
II.B	CatBoost	0.792	0.741	0.8
II.C	XGBoost	0.753	0.691	0.740
II.D	CatBoost	0.778	0.72	0.8
**II.E**	**GB**	**0.85**	**0.814**	**0.844**
III.A	LSTM	0.744	0.696	0.726
III.B	LSTM	0.742	0.679	0.787
**III.C**	**LSTM**	**0.805**	**0.759**	**0.843**

The best-performing models are highlighted in bold. Dataset I.A: baseline radiomic features, dataset I.B: baseline radiomic features and baseline PSA, dataset I.C: baseline radiomic features and baseline PSAd, dataset II.A: delta-radiomic features, dataset II.B: delta-radiomic features and delta-PSA, dataset II.C: delta-radiomic features and delta-PSAd, dataset II.D: delta-radiomic features and final PSA, dataset II.E: delta-radiomic features and final PSAd, dataset III.A: time series of radiomic features, dataset III.B: time series of radiomic features and time series of PSA, dataset III.C: time series of radiomic features and time series of PSAd.

**Table 3:** Performance of models built on an optimal subset of important features for predicting histopathological PCa progression on AS. Classification quality metrics were calculated using the LOOCV scheme.

Dataset, Model	Number of Optimal Features	Balanced Accuracy	F1-Score	AUC
I.C, GB	7	0.76	0.702	0.793
II.E, GB	17	0.865	0.836	0.913
III.C, LSTM	7	0.861	0.828	0.917

Dataset I.C: baseline radiomic features and baseline PSAd, dataset II.E: delta-radiomic features and final PSAd, dataset III.C: time series of radiomic features and time series of PSAd.

**Table 4:** Sensitivity and specificity analysis of the resulting models for predicting histopathological PCa progression on AS. Classification quality metrics were calculated using the LOOCV scheme.

Dataset, Model, # Features	Sensitivity	Specificity	PPV	NPV
I.C, GB, 7	0.690	0.830	0.714	0.813
II.E, GB, 17	0.793	**0.936**	**0.885**	0.88
III.C, LSTM, 7	**0.828**	0.894	0.828	**0.894**

Values with the best performance across the three models are highlighted in bold. Dataset I.C: baseline radiomic features and baseline PSAd, dataset II.E: delta-radiomic features and final PSAd, dataset III.C: time series of radiomic features and time series of PSAd, PPV: Positive Predictive Value, NPV: Negative Predictive Value.

## Data Availability

The raw data supporting the conclusions of this article will be made available by the authors on request.

## Supplementary Materials

The following supporting information can be downloaded at: https://www.mdpi.com/article/10.3390/cancers17223598/s1, Supplementary Table S1: MRI acquisition parameters; Supplementary Table S2: Final radiomic feature set following robustness analysis; Supplementary Table S3: Tunable hyperparameters of machine learning models; Supplementary Table S4: Performance of models for predicting histopathological progression of prostate cancer on active surveillance using baseline features; Supplementary Table S5: Performance of models for predicting histopathological progression of prostate cancer on active surveillance using delta features; Supplementary Figure S1: Average influence (mean absolute SHAP values) of features on the model predictions based on baseline radiomic features and baseline PSAd; Supplementary Figure S2: Average influence (mean absolute SHAP values) of features on the model predictions based on delta-radiomic features and final PSAd; Supplementary Figure S3: Average influence (mean absolute SHAP values) of features on the model predictions based on time series of radiomic features and time series of PSAd. References [70,71,72] are cited in the supplementary materials.
