# Supplementary material for: Improving the Potential for Predicting Prostate Cancer Progression in Patients on Active Surveillance Using Explainable Artificial Intelligence
Source: Cancers (Basel). 2025 Nov 7;17(22):3598. doi: 10.3390/cancers17223598 (PMC12650280; doi:10.3390/cancers17223598)
Supplement: Supplementary file 1 [file cancers-17-03598-s001.zip › cancers-3942966-supplementary.pdf]

Improving the Potential for Predicting Prostate Cancer Progression in Patients on Active Surveillance Using Explainable Artificial Intelligence

Supplementary Materials

Supplementary Table S1. MRI acquisition parameters. TR: repetition time, TE: echo time, FOV: field of view, DCE: dynamic contrast enhancement, FSE: fast spin echo, DWI: diffusion-weighted imaging, LAVA: liver acquisition with volume acceleration. * Denote sequences used as part of the biparametric protocol.

**Parameter**	**Localiser***	**Axial T1 FSE***	**Axial T2 FSE***	**Sagittal T2 FSE**	**Axial DWI***	**Axial DWI Focus**	**DCE LAVA**
TE/TR, ms	20/200	30/789	102/3743	102/3743	85/3775	60/4000	min full/4.3
FOV, cm	30	32	18	22	28	24	24
Matrix	256	512	384	288	128	356	192
Slice thickness, mm	3	6	3	1	3	3	3
Gap, mm	0	2	0	0	0	0	0
Phase	128	320	224	224	128	80	192
b-values	–	–	–	–	100, 750, 1400	100, 2000	–
Synthetic b-values	–	–	–	–	2000, 2500	2500	–
Scan time, min	00:35	02:32	05:22	03:13	02:42	04:52	06:22

Supplementary Table S2. Final radiomic feature set following robustness analysis. T2WI: T2-weighted imaging, ADC: apparent diffusion coefficient. Feature description can be found in the online PyRadiomics documentation (https://pyradiomics.readthedocs.io/en/latest/).

**T2WI-derived features**	**ADC-derived features**
First-order	***First-order***
10th Percentile	10th Percentile
90th Percentile	90th Percentile
Energy	Energy
Interquartile Range	Interquartile Range
Maximum	Maximum
Mean	Mean
Mean Absolute Deviation	Mean Absolute Deviation
Median	Median
Minimum	Minimum
Robust Mean Absolute Deviation	Range
Root Mean Squared	Robust Mean Absolute Deviation
Total Energy	Root Mean Squared
Variance	Total Energy
Gray Level Co-occurrence Matrix (GLCM)	Variance
Correlation	***Gray Level Co-occurrence Matrix (GLCM)***
IMC1 (Informational Measure of Correlation 1)	IMC1 (Informational Measure of Correlation 1)
Gray Leven Size Zone Matrix (GLSZM)	Joint Entropy
Size Zone NonUniformity	***Gray Level Dependence Matrix (GLDM)***
Shape	Dependence NonUniformity
Maximum 2D Diameter Row	***Gray Level Run Length Matrix (GLRLM)***
	Run Length NonUniformity
	Gray Leven Size Zone Matrix (GLSZM)
	Size Zone NonUniformity
	Neighboring Gray-Tone Difference Matrix (NGTDM)
	Strength
	Shape
	Flatness
	Least Axis Length
	Maximum 2D Diameter Row
	Mesh Volume
	Minor Axis Length
	Surface Area
	Voxel Volume

Supplementary Table S3. Tunable hyperparameters of machine learning models. kNN: k-Nearest Neighbors, LR: Logistic Regression, SVM: Support Vector Machine, DT: Decision Tree, RF: Random Forest, GB: Gradient Boosting, XGBoost: eXtreme Gradient Boosting, LightGBM: Light Gradient Boosted Machine, CatBoost: Category Boosting, LSTM: Long Short-Term Memory.

**Model**	**Hyperparameter**	**Description**	**Search Distribution**
kNN	n_neighbors	Number of neighbors	int (low=1, high=35, step=1)
LR (penalty=l2, solver=liblinear)	C	Inverse of regularization strength	float uniform (low= 0.00001, high=1.0)
SVM (kernel=linear)	C	Regularization parameter	float uniform (low= 0.00001, high=1.0)
DT	max_depth	The maximum depth of the tree	[2, 3, 4, 5]
	min_samples_split	The minimum number of samples required to split an internal node	int (low=2, high=20, step=1)
	min_samples_leaf	The minimum number of samples required to be at a leaf node	int (low=1, high=10, step=1)
RF	n_estimators	The number of trees in the forest	int (low=5, high=50, step=1)
	max_depth	The maximum depth of the tree	[2, 3, 4, 5]
	min_samples_split	The minimum number of samples required to split an internal node	int (low=2, high=20, step=1)
	min_samples_leaf	The minimum number of samples required to be at a leaf node	int (low=1, high=10, step=1)
	max_features	The number of features to consider when looking for the best split	[0.1, 0.2, 0.3, 0.4, 0.5]
GB	learning_rate	Learning rate shrinks the contribution of each tree by learning_rate	float uniform (low= 0.00001, high=0.5)
	n_estimators	The number of boosting stages to perform	int (low=5, high=50, step=1)
	max_depth	The maximum depth limits the number of nodes in the tree	[2, 3, 4, 5]
	min_samples_split	The minimum number of samples required to split an internal node	int (low=2, high=20, step=1)
	min_samples_leaf	The minimum number of samples required to be at a leaf node	int (low=1, high=10, step=1)
XGBoost	n_estimators	Number of boosting rounds	int (low=5, high=50, step=1)
	max_depth	Maximum tree depth for base learners	[2, 3, 4, 5]
	learning_rate	Boosting learning rate	float uniform (low=0.00001, high=0.5)
	gamma	Minimum loss reduction required to make a further partition on a leaf node of the tree	float uniform (low=0.0, high=5.0)
	min_child_weight	Minimum sum of instance weight (hessian) needed in a child	int (low=1, high=10, step=1)
	colsample_bytree	Subsample ratio of columns when constructing each tree	[0.7, 0.8, 0.9, 1.0]
LightGBM	n_estimators	Number of boosted trees to fit	int (low=5, high=50, step=1)
	learning_rate	Boosting learning rate	float uniform (low=0.00001, high=0.5)
	max_depth	Maximum tree depth for base learners	[2, 3, 4, 5]

	num_leaves	Maximum tree leaves for base learners	int (low=10, high=31, step=1)
	colsample_bytree	Subsample ratio of columns when constructing each tree	[0.7, 0.8, 0.9, 1.0]
	min_data_in_leaf	Minimal number of data in one leaf	int (low=3, high=10, step=1)
CatBoost (grow_policy= SymmetricTree)	n_estimators	The maximum number of trees that can be built when solving machine learning problems	int (low=5, high=50, step=1)
	learning_rate	The learning rate	float uniform (low=0.00001, high=0.5)
	depth	Depth of the trees	[2, 3, 4, 5]
	l2_leaf_reg	Coefficient at the L2 regularization term of the cost function	float uniform (low=1.0, high=5.0)
	colsample_bylevel	The percentage of features to use at each split selection, when features are selected over again at random	[0.7, 0.8, 0.9, 1.0]
LSTM	lstm_units	The ith element represents the number of neurons in the ith layer	[[8], [16], [32], [64], [16, 8]]
	dropout	Fraction of the units to drop for the linear transformation of the inputs	float uniform (low=0.0, high=0.5)
	recurrent_dropout	Fraction of the units to drop for the linear transformation of the recurrent state	float uniform (low=0.0, high=0.5)
	learning_rate	The learning rate	float uniform (low=0.00001, high=0.5)
	epochs	Number of epochs to train the model	[25, 50, 75, 100, 125]
	batch_size	Number of samples per gradient update	[4, 8, 16]

Supplementary Table S4. Performance of models for predicting histopathological progression of prostate cancer on active surveillance using baseline features. Classification quality metrics were calculated using a leave-one-out cross-validation (LOOCV) scheme. The best-performing models are highlighted in bold. PSA: Prostate-Specific Antigen, PSAd: PSA density, AUC: Area Under the Receiver Operating Characteristic Curve, kNN: k-Nearest Neighbors, LR: Logistic Regression, SVM: Support Vector Machine, DT: Decision Tree, RF: Random Forest, GB: Gradient Boosting, XGBoost: eXtreme Gradient Boosting, LightGBM: Light Gradient Boosted Machine, CatBoost: Category Boosting.

Dataset	Model	Balanced accuracy	F1-score	AUC
I.A: baseline radiomic features	kNN	0.567	0.341	0.575
	LR	0.604	0.551	0.588
	SVM	0.558	0.436	0.029
	DT	0.605	0.458	0.583
	RF	0.610	0.517	0.574
	GB	0.646	0.538	0.665
	**XGBoost**	**0.695**	**0.600**	**0.623**
	LightGBM	0.648	0.561	0.639
	CatBoost	0.693	0.621	0.661
I.B: baseline radiomic features and baseline PSA	kNN	0.586	0.473	0.570
	LR	0.615	0.559	0.600
	SVM	0.519	0.393	0.182
	DT	0.606	0.525	0.571
	RF	0.607	0.491	0.599
	GB	0.635	0.528	0.653
	XGBoost	0.646	0.538	0.635
	LightGBM	0.620	0.526	0.608
	**CatBoost**	**0.659**	**0.571**	**0.662**
I.C: baseline radiomic features and baseline PSAd	kNN	0.571	0.475	0.571
	LR	0.621	0.571	0.635
	SVM	0.594	0.449	0.578
	DT	0.697	0.618	0.627
	RF	0.663	0.566	0.594
	**GB**	**0.719**	**0.642**	**0.704**
	XGBoost	0.712	0.627	0.644
	LightGBM	0.628	0.510	0.645
	CatBoost	0.652	0.556	0.680

Supplementary Table S5. Performance of models for predicting histopathological progression of prostate cancer on active surveillance using delta features. Classification quality metrics were calculated using a leave-one-out cross-validation (LOOCV) scheme. The best-performing models are highlighted in bold. PSA: Prostate-Specific Antigen, PSAd: PSA density, AUC: Area Under the Receiver Operating Characteristic Curve, kNN: k-Nearest Neighbors, LR: Logistic Regression, SVM: Support Vector Machine, DT: Decision Tree, RF: Random Forest, GB: Gradient Boosting, XGBoost: eXtreme Gradient Boosting, LightGBM: Light Gradient Boosted Machine, CatBoost: Category Boosting.

Dataset	Model	Balanced accuracy	F1-score	AUC
II.A: delta-radiomic features	kNN	0.669	0.582	0.715
	LR	0.698	0.647	0.731
	SVM	0.655	0.576	0.621
	DT	0.646	0.538	0.563
	RF	0.742	0.679	0.767
	GB	0.753	0.691	0.769
	XGBoost	0.756	0.700	0.759
	**LightGBM**	**0.764**	**0.704**	**0.817**
	CatBoost	0.760	0.702	0.785
II.B: delta-radiomic features and delta-PSA	kNN	0.693	0.621	0.722
	LR	0.679	0.613	0.665
	SVM	0.654	0.522	0.700
	DT	0.646	0.538	0.570
	RF	0.764	0.704	0.786
	GB	0.764	0.704	0.764
	XGBoost	0.756	0.700	0.752
	LightGBM	0.770	0.714	0.767
	**CatBoost**	**0.792**	**0.741**	**0.800**
II.C: delta-radiomic features and delta-PSAd	kNN	0.680	0.593	0.727
	LR	0.692	0.636	0.730
	SVM	0.620	0.526	0.598
	DT	0.646	0.538	0.570
	RF	0.747	0.679	0.786
	GB	0.742	0.679	0.781
	**XGBoost**	**0.753**	**0.691**	**0.740**
	LightGBM	0.744	0.667	0.778
	CatBoost	0.729	0.654	0.720
II.D: delta-radiomic features and final PSA	kNN	0.678	0.571	0.731
	LR	0.709	0.657	0.735
	SVM	0.664	0.533	0.716
	DT	0.687	0.607	0.612
	RF	0.725	0.655	0.729
	GB	0.747	0.679	0.775
	XGBoost	0.742	0.679	0.786
	LightGBM	0.742	0.679	0.799
	**CatBoost**	**0.778**	**0.720**	**0.800**
II.E: delta-radiomic features and final PSAd	kNN	0.695	0.600	0.744
	LR	0.709	0.657	0.742
	SVM	0.669	0.582	0.685
	DT	0.672	0.600	0.614
	RF	0.826	0.786	0.820
	**GB**	**0.850**	**0.814**	**0.844**
	XGBoost	0.809	0.764	0.866
	LightGBM	0.847	0.815	0.868
	CatBoost	0.809	0.764	0.842

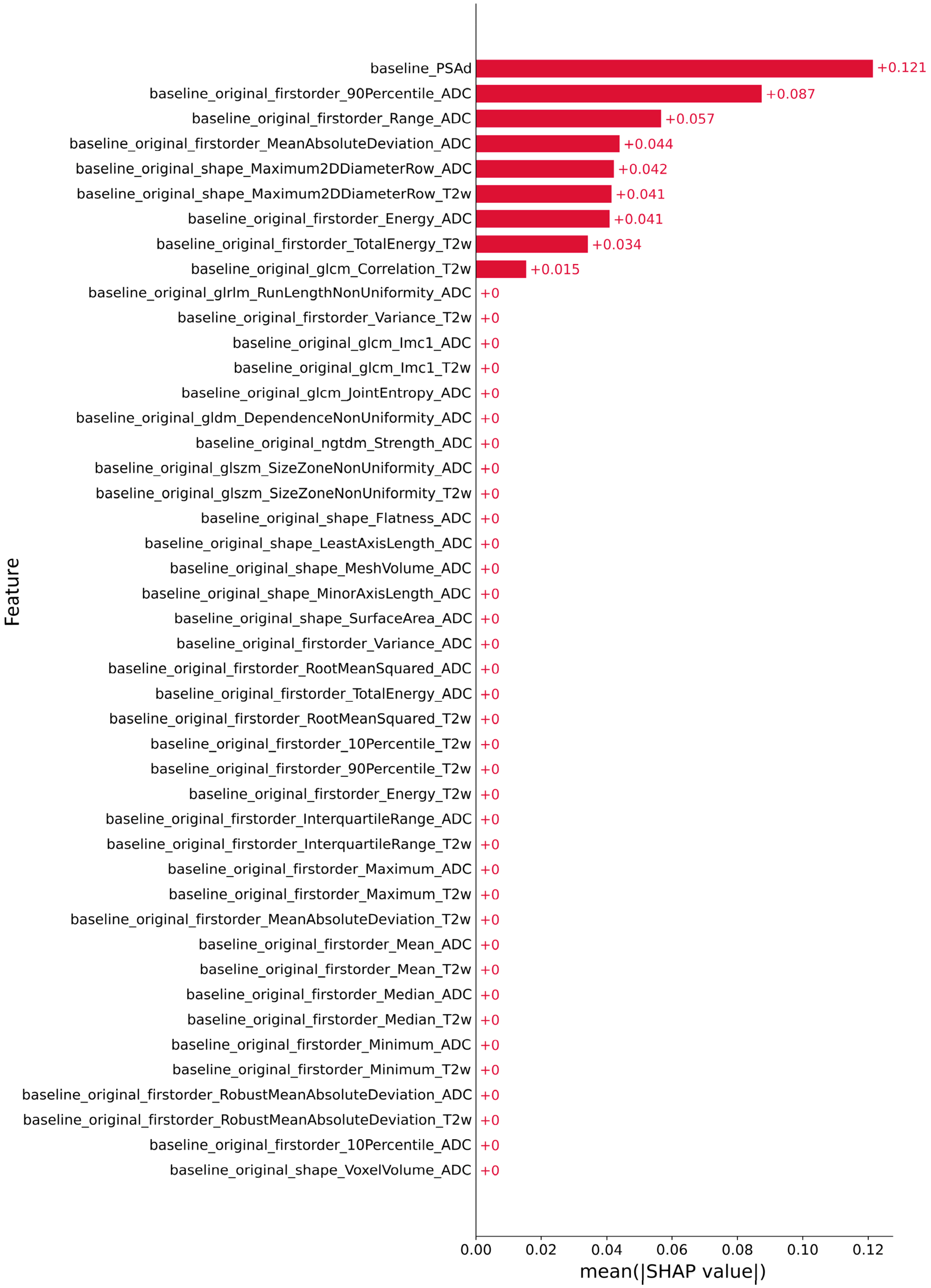

Supplementary Figure S1. Average influence (mean absolute SHAP values) of features on the model predictions based on baseline radiomic features and baseline PSAd. Features are sorted by descending importance (top: most important).

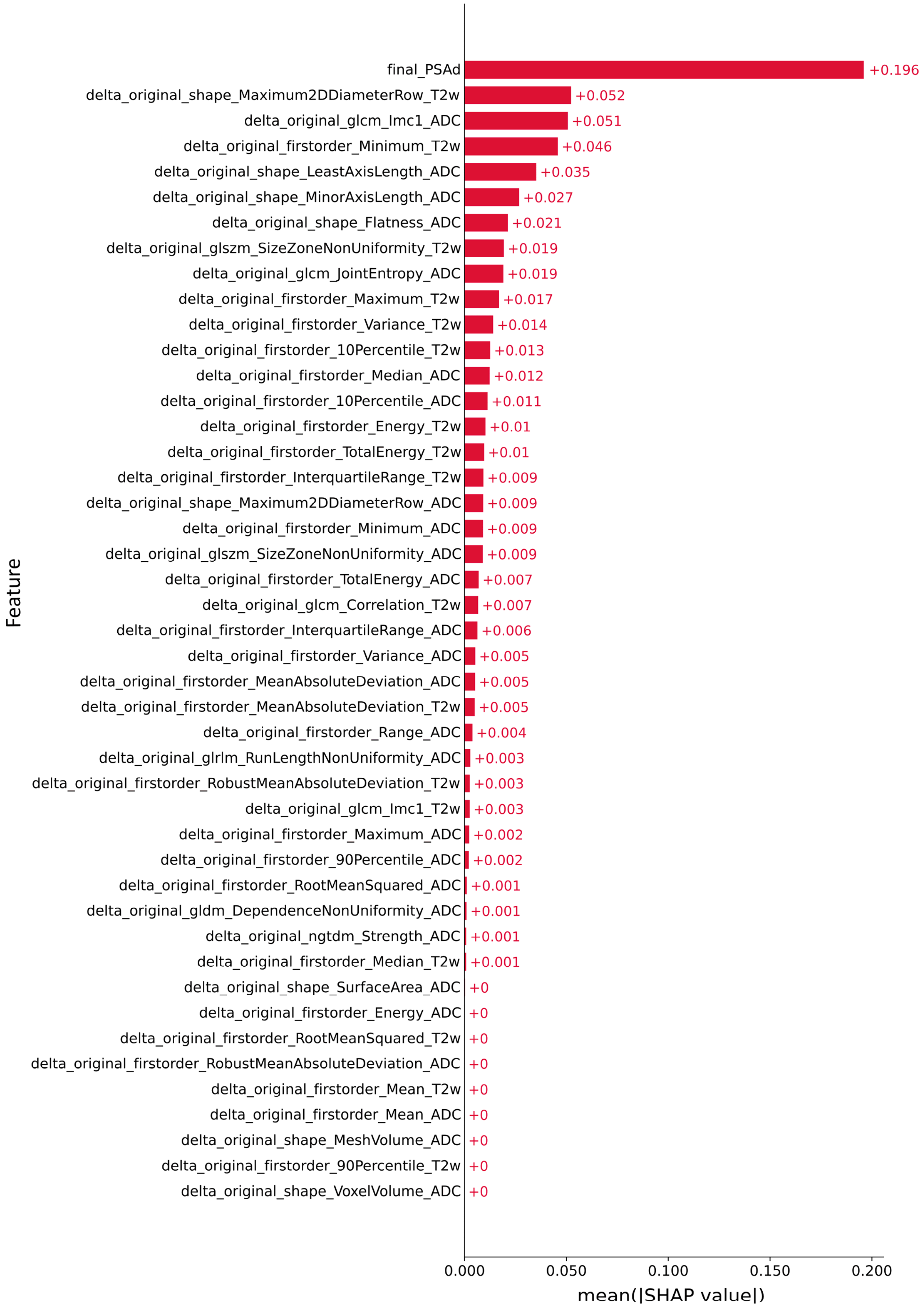

Supplementary Figure S2. Average influence (mean absolute SHAP values) of features on the model predictions based on delta-radiomic features and final PSAd. Features are sorted by descending importance (top: most important).

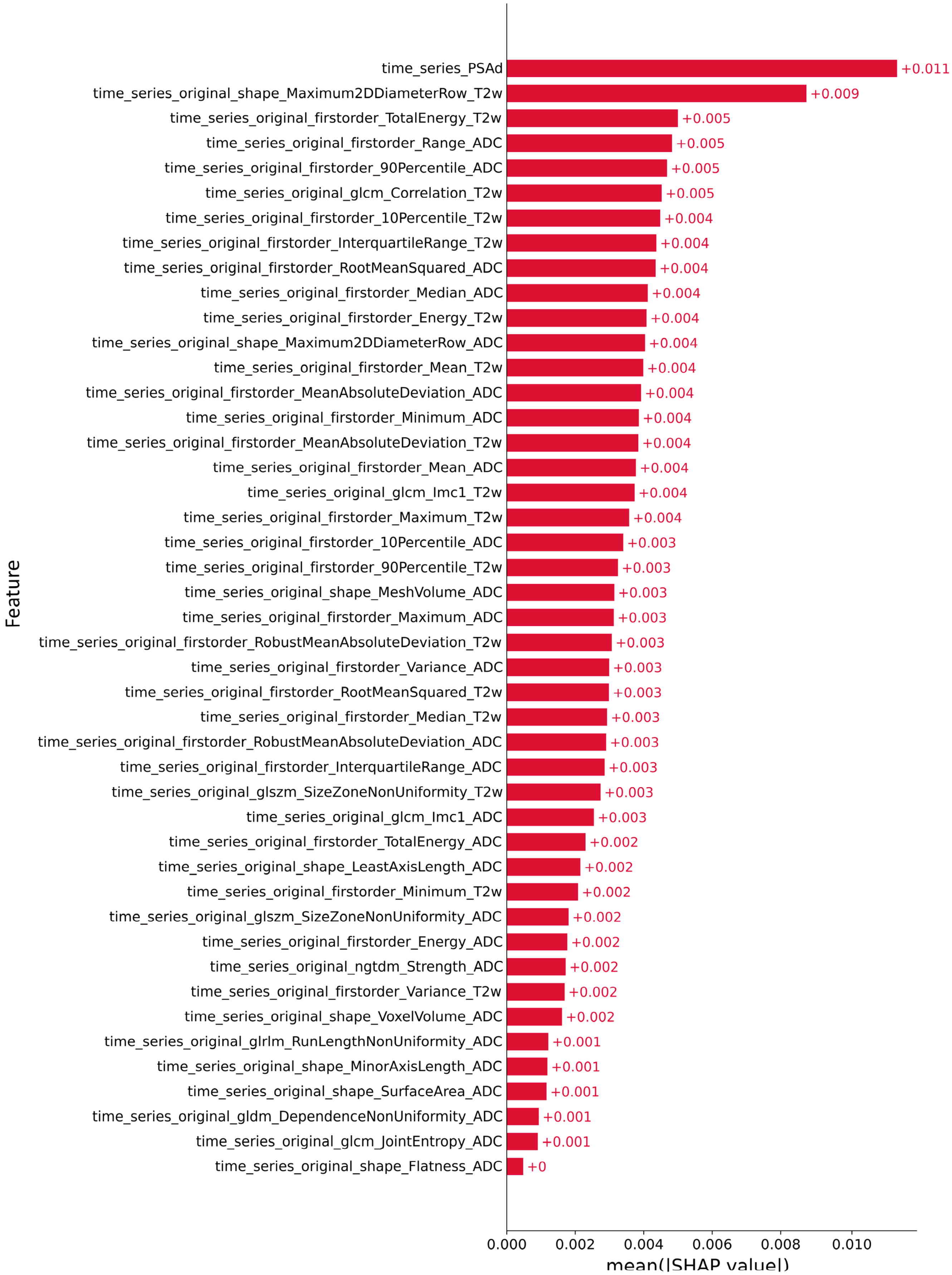

Supplementary Figure S3. Average influence (mean absolute SHAP values) of features on the model predictions based on time series of radiomic features and time series of PSAd. Features are sorted by descending importance (top: most important).

Robustness analysis of radiomic features

The extracted features were considered highly robust if they remained unchanged following the application of ROI perturbations and were independent from the MRI acquisition parameters.

T2WI- and ADC-derived ROIs were perturbed by using morphological operators (i.e. opening and closing with a 3D spherical structuring element of 1-pixel radius). Morphological perturbations of ROIs were performed using the *Scipy.ndimage.morphology* functions (*binary_opening* and *binary_closing*) in the SciPy v1.3.2 multidimensional image processing package in Python v3.7. We thereby produced three versions of each ROI: original, opening, and closing. This procedure tests the variability of ROIs by emulating the intra- and inter-reader dependence of manual contouring [1]. Starting from these three sets of ROIs, the radiomic features were extracted for both time-points and MRI sequences using different quantisation configurations: the number of bins varied in $\{8, 16, 32, 64, 128, 256\}$. Thus, the two perturbations applied to the original ROIs and the different quantization settings yielded 18 configurations of radiomic features for each sequence and time-point pair.

The ICC was considered to determine the most robust features against the ROI perturbations whilst the number of bins varied too [2]. ICC analysis was applied to these 18 configurations of features for identifying the number of bins that achieved the largest set of highly robust features extracted separately on T2WI and ADC. In particular, we considered the two-way random-effects model (or mixed-effects), consistency, single rater/measurement, $ICC(3,1)$ [3]:

$$ICC(3,1) = \frac{MS_R - MS_E}{MS_R + (k-1)\,MS_E},$$

where MS_R and MS_E are the mean square for rows and mean square for error, respectively. The cutoff value was set to 0.8.

The chosen number of bins (128) represents the most reliable quantisation configuration (i.e. rebinning) according to the ROI perturbations *via* morphological operators. A cut-off value of 0.8 was used for the ICC to identify the number of features with high robustness. The used quantisation configuration was selected by considering the number of bins that obtained the highest number of robust features for T2w and ADC at both time-points. The highly robust features with ICC > 0.8 were then used in the downstream pre-processing phases.

Secondly, the extracted features might be affected by the MRI acquisition characteristics, such as scanner type, scanner settings, imaging protocols and acquisition parameters [3]. In this study, we calculated the Spearman correlation coefficient for each radiomic feature against the following MRI acquisition parameters: (*i*) echo time (TE); (*ii*) repetition time (TR); (*iii*) flip angle; (*iv*) slice thickness; (*v*) spacing between slices; (*vi*) pixel spacing. Importantly, Spearman's correlation analysis showed no relationship between any radiomic features and MRI acquisition parameters (P > 0.05 for all with no multiplicity correction applied).

Only features that were robust across all time points for all patients were included in the predictive modeling.

1. Cattell, R.; Chen, S.; Huang, C. Robustness of radiomic features in magnetic resonance imaging: review and a phantom study. *Vis. Comput. Ind. Biomed. Art* **2019**, *2*, 19. https://doi.org/10.1186/s42492-019-0025-6.
2. Scalco, E.; Belfatto, A.; Mastropietro, A.; Rancati, T.; Avuzzi, B.; Messina, A.; Valdagni, R.; Rizzo, G. T2w-MRI signal normalization affects radiomics features reproducibility. *Med. Phys.* **2020**, *47*, 1680–1691. https://doi.org/10.1002/mp.14038.
3. Přibil, J.; Přibilová, A.; Frollo, I. Analysis of the influence of different settings of scan sequence parameters on vibration and noise generated in the open-air MRI scanning area. *Sensors* **2019**, *19*, 4198. https://doi.org/10.3390/s19194198.
